# Survival from Maternal Cardiac Arrest Complicating Coronavirus Disease 2019

**DOI:** 10.1155/2021/3762198

**Published:** 2021-07-19

**Authors:** Andrea D. Shields, John J. Byrne, Meghan Munisteri, Michael Wood

**Affiliations:** ^1^Department of Obstetrics and Gynecology, University of Connecticut, Farmington, Connecticut, USA; ^2^Department of Obstetrics and Gynecology, Baylor College of Medicine, San Antonio, Texas, USA; ^3^Department of Obstetrics and Gynecology, Brooke Army Medical Center, San Antonio, Texas, USA; ^4^Department of Medicine, Shannon Medical Center, San Angelo, Texas, USA

## Abstract

**Introduction:**

Coronavirus disease 2019 (COVID-19) has been linked to significant cardiovascular complications such as cardiac arrest, which are associated with a poor prognosis in adults. Little is known about the cardiac complications, specifically cardiac arrest, of COVID-19 during pregnancy and postpartum periods.

**Case:**

We present a case of survival and full neurological recovery after maternal cardiac arrest associated with COVID-19 in a postpartum female. Her postpartum course was also associated with seizures attributed to posterior reversible encephalopathy syndrome. After 19 days in the hospital, she was discharged home neurologically intact.

**Conclusion:**

More information is needed to determine the range of short- and long-term cardiac complications that may be associated with COVID-19 during pregnancy and postpartum. Additionally, pregnant patients with COVID-19 may be more likely to survive cardiac arrest compared to the general population.

## 1. Introduction

Cardiac complications associated with coronavirus disease 2019 (COVID-19) have been well described [1–4]. Up to one-third of nonpregnant adults with COVID-19 that are admitted to the intensive care unit (ICU) develop myocardial injury [1], associated with an increased risk of mortality [1, 2]. Additionally, long-term cardiovascular dysfunction has been reported during the convalescent phase in survivors of COVID-19 [5–7]. Not surprisingly, cardiac complications from COVID-19 are more likely to occur in older adults and those with underlying comorbidities [8, 9]. However, there is limited information on cardiac complications in pregnant and postpartum reproductive-age females. We present a case of maternal cardiac arrest associated with COVID-19, complicated by sinus node dysfunction requiring a permanent pacemaker. This is the only case of survival from COVID-19 associated maternal cardiac arrest that has been reported in the literature to date.

## 2. Case Presentation

Written consent was obtained from the patient as there is identifiable data included in this case report.

**Figure 1:**
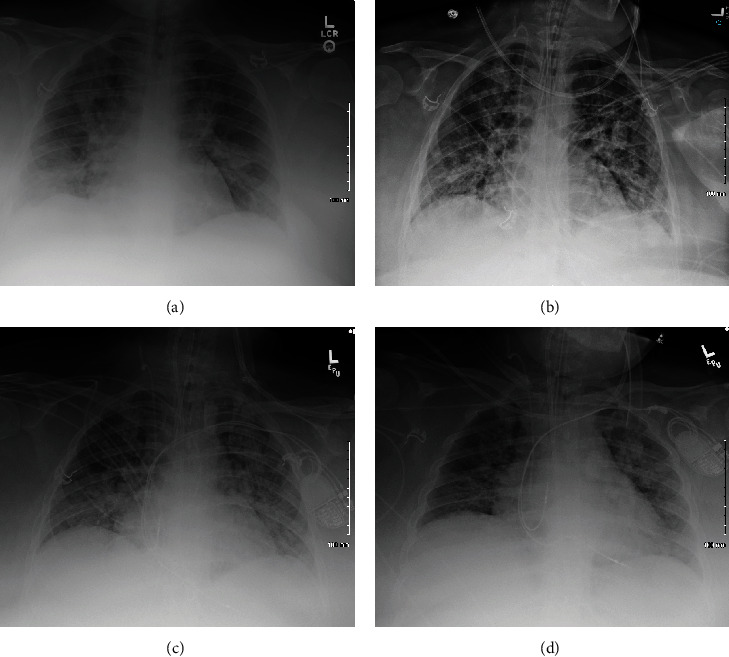
Progression of lung findings on chest radiography in postpartum patient with severe COVID 19.

A 28-year-old Hispanic female gravida 2 para 1001 at 38+6 weeks' gestation with pregnancy complicated by class III obesity (BMI 60), mixed anxiety and depressive disorder, and Rh negative status presented to the emergency department (ED) with a 5-day history of cough, congestion, fevers, and shortness of breath. Her vitals on presentation were blood pressure 124/65 mmHg, pulse 131 beats per minute, respiratory rate of 24 breaths per minute, oxygen saturation of 95% on room air, and temperature of 38.7 Celsius. She appeared uncomfortable with difficulty breathing; her physical exam revealed clear lungs bilaterally, tachycardia, and reassuring fetal heart rate tracing. Laboratory evaluation revealed an elevated CRP, AST, procalcitonin, and low vitamin D level. A chest radiograph demonstrated bilateral pulmonary infiltrates (see Figure 1(a)). Blood cultures were obtained. A nasopharyngeal swab for SARS-CoV-2 PCR was obtained and returned positive, and she was admitted to the hospital for treatment of moderate COVID-19. Within 12 hours of presenting to the ED, she required 6 L by nasal cannula (NC) to maintain her oxygen saturation > 92%, and a decision was made to proceed with expedited delivery via cesarean section due to her worsening respiratory status. Intravenous remdesivir and dexamethasone were initiated preoperatively. She underwent an uncomplicated primary low transverse cesarean section productive of a female infant weighing 3325 g.

Postoperatively, the patient was improving with a lower supplemental oxygen requirement and decreased work of breathing. On POD#3, her oxygen requirements increased rapidly from supplemental oxygen via nasal cannula up to high-flow nasal cannula oxygen with 100% FiO2. She deteriorated further with increased work of breathing and significant hypoxemia and was placed on Bilevel Positive Airway Pressure (BiPAP). A chest radiograph demonstrated interval worsening of bilateral diffuse airspace opacities (see Figure 1(b)). While on BiPAP, she continued to have significant hypoxemia with worsening tachypnea and tachycardia; therefore, she was transferred to the ICU for acute respiratory failure with severe acute respiratory distress syndrome (ARDS), and rapid sequence intubation was performed.

On the ventilator, the patient developed worsening hypoxemia, at which time the endotracheal tube was repositioned and a chest radiograph confirmed correct positioning also demonstrating bilateral pulmonary infiltrates (see Figure 1(c)). She continued to desaturate and developed bradycardia and then pulseless electrical activity (PEA) arrest. Cardiopulmonary resuscitation (CPR) was initiated immediately. Return of spontaneous circulation was achieved after 5 minutes of CPR and a dose of IV epinephrine. The patient was placed on the ventilator to maintain her oxygen saturation ≥ 90%. Chest radiograph revealed interval changes including mild enlargement of the cardiac silhouette, pulmonary edema and/or inflammatory infiltrates, and a right pleural effusion (see Figure 1(d)). She was started on low dose pressor support for hypotension. On POD#5, she developed sinus pauses and periods of sinus arrest, and cardiology and electrophysiology consults were requested. Echocardiogram demonstrated normal left ventricular function with an ejection fraction of 60-65%, a mildly dilated left atrium, mild tricuspid valve regurgitation, and moderately elevated pulmonary systolic pressure with pulmonary artery systolic pressure of 50 mmHg. There was no evidence of myocarditis or sepsis (i.e., blood cultures from admission were negative). A temporary pacer was placed followed by a permanent dual chamber pacemaker on POD#6. She was weaned off vasopressor support and down to minimal ventilator settings by POD#10, with intermittent nonsustained hypertension with a maximal blood pressure of 165/118 mmHg.

The patient developed a generalized myoclonic-tonic seizure during a trial of spontaneous breathing (TSB) concerning for eclampsia. She was started on IV magnesium therapy for presumed eclampsia complicated by posterior reversible encephalopathy syndrome (PRES) versus COVID encephalopathy. This was subsequently comanaged by consultants in Neurology and Maternal Fetal Medicine. After several days of seizure management with levetiracetam and lacosamide every 12 hours and the development of encephalopathy syndrome, the patient was weaned from sedation and passed a TSB and was successfully extubated after remaining mechanically ventilated for a total of 11 days. The patient had no further seizure activity and was discharged home 4 days later (POD#18) on 1 L of oxygen and with home health care services. She was discharged home on 200 mg lacosamide QD, 500 mg levetiracetam BID, and 81 mg aspirin QD with outpatient neurology and electrophysiology follow-up arranged.

## 3. Discussion

This case describes severe cardiac morbidity and potential for mortality associated with a young, relatively healthy pregnant woman with critical COVID-19. Cardiovascular complications have been identified in nonpregnant individuals—myocarditis, myocardial injury, acute myocardial infarction, cardiac arrest, dysrhythmias, heart failure, and venous thromboembolism [1–4]. However, myocardial injury in pregnancy and the postpartum period are limited to case series. Of the 154 pregnant women admitted to one tertiary care hospital in Dominican Republic for severe COVID-19, 15 patients (10%) developed evidence of myocardial injury [4]. All of these individuals had abnormal laboratory studies and imaging consistent with myocardial injury. After admission, maternal deaths occurred in 2 of the 15 patients. Long-term cardiac dysfunction has also been reported in nonpregnant individuals [6, 7]; however, we are unable to locate any case reports of long-term cardiac dysfunction during the convalescent phase in survivors of COVID-19 during pregnancy or postpartum.

One of the most concerning cardiovascular complications associated with COVID-19 is cardiac arrest. Recent publications suggest a 100% mortality rate from in-hospital cardiac arrest in the setting of COVID-19, and some hospitals have considered universal do-not-resuscitate orders for these patients [8, 9]. Shah and colleagues studied 1,094 patients hospitalized for COVID-19 between March and August 2020 [8]. Of the 63 patients that suffered from in-hospital cardiac arrest (IHCA) with attempted resuscitation, the most common rhythms were pulseless electrical activity and asystole. Although return of spontaneous circulation was achieved in 29% patients, it was brief in all of them, and the in-hospital mortality was 100%. Several risk factors for COVID-19 cardiac arrest also emerged, including older age, male sex, African American race, comorbidities (hypertension, obesity, diabetes, and chronic kidney disease), and a hospital course complicated by development of septic shock, ARDS, mechanical ventilation, dialysis, vasopressors use, or admission to the ICU.

Similar findings were noted by Thapa et al. who studied 1309 patients hospitalized with COVID-19 between March and April 2020 [9]. Risk factors were similar to those reported by Shah and colleagues. The in-hospital mortality rate for this cohort was 100%. To our knowledge, none of the patients in these case series were pregnant or postpartum, and this is the only case we were able to locate that describes survival to discharge with complete neurological recovery from PEA arrest complicating COVID-19 in the puerperium.

Limited evidence suggests that pregnant women have significantly better odds of surviving non-trauma-related cardiac arrest than nonpregnant women, but the reasons for this observation are not entirely clear [10, 11]. Nonetheless, the majority of pregnant and postpartum patients with COVID-19 will be younger and have less comorbidities compared with nonpregnant adults with COVID-19 and may benefit from aggressive cardiopulmonary resuscitation in this setting. Until more information is known, healthcare workers should be aware of the potential significant cardiac complications that may occur in critically ill pregnant and postpartum patients with COVID-19.

## Data Availability

The data collected for this case contains protected personal health information. This information was retrieved after obtaining written informed consent from the patient discussed in this paper. Thus, we are unable to freely provide access to our data for patient privacy reasons.

## Additional Points

*Précis*. COVID-19 in pregnant and postpartum patients can result in devastating cardiac complications.
